# Profile and experiences during the incarceration of transgender women and travestis (TGW) in Brazil: a cross-sectional study

**DOI:** 10.1590/1980-549720240014.supl.1

**Published:** 2024-08-19

**Authors:** Andréa Fachel Leal, Cristine Coelho Cazeiro, Ana Carolina Einsfeld Mattos, Bruna Hentges, Luciana Barcellos Teixeira, Daniela Riva Knauth, Laio Magno, Inês Dourado, Maria Amélia de Sousa Mascena Vera

**Affiliations:** I Universidade Federal do Rio Grande do Sul, Department of Sociology – Porto Alegre (RS), Brazil.; II Universidade Federal do Rio Grande do Sul, Graduate Program in Epidemiology – Porto Alegre (RS), Brazil.; III Universidade Federal do Rio Grande do Sul, Graduate Program in Public Policies – Porto Alegre (RS), Brazil.; IV Universidade Federal do Rio Grande do Sul, Department of Collective Health – Porto Alegre (RS), Brazil.; V Universidade Federal do Rio Grande do Sul, Department of Social Medicine – Porto Alegre (RS), Brazil.; VI Universidade do Estado da Bahia – Salvador (BA), Brazil.; VII Universidade Federal da Bahia – Salvador (BA), Brazil.; VIII Santa Casa de São Paulo, School of Medical Sciences – São Paulo (SP), Brazil.

**Keywords:** Transgender persons, Travestis, Prisoners, Prisons, Social vulnerability

## Abstract

**Objective:**

The objective of the present study is to describe the sociodemographic and behavioral characteristics of a group of *transgender women and travestis (TGW)* with a history of incarceration and the institutional and social context of this experience in Brazil.

**Methods:**

The analyzed data were derived from the TransOdara Study, a cross-sectional study conducted in five Brazilian capitals from December 2019 to July 2021. Participants were recruited using the Respondent-Driven Sampling (RDS) technique, in which, after an initial formative and exploratory stage, the first participants were identified; in turn, these participants recruited up to six other *transgender women and travestis* for the research. The study’s outcome was the experience of incarceration throughout life, captured through the question: “Have you ever been arrested in your life?”

**Results:**

A total of 1,245 TGW were interviewed, of which 20.3% (n=253) experienced incarceration. Incarceration was more frequent among those aged 33 to 42 years (35.6%), with lower level of education (45.5%, p<0.001), engaged in informal work (30.3%), without a partner (67.2%), and among those who reported illicit drug use (66.4%). The majority (60.9%) of TGW were incarcerated with cisgender men, and the most common reasons for imprisonment were drug trafficking (30.4%) followed by robbery (29.2%). Over a quarter of the interviewees (26.3%) experienced assault, and 13.8% reported experiencing sexual violence during incarceration.

**Conclusion:**

The results emphasize the high prevalence of incarceration among TGW. This incarceration takes place in male wards and in a context of high rates of physical and sexual violence.

## INTRODUCTION

Transgender people suffer various forms of discrimination and gender-based violence, which restrict their access to resources necessary to achieve and maintain a dignified and healthy life, such as education, employment, and income^1,2^. Consequently, transgender individuals often find themselves involved in informal activities, such as sex work, or even illicit activities such as drug trafficking^3^.

The stigma and consequent social exclusion of transgender people impact incarceration rates and the way they are treated in the criminal justice system, causing the incarceration rates of transgender individuals to be higher than those of the general population^4,5^. The experience of incarceration for transgender people leads to a maximization of the violations suffered in freedom: physical, psychological, and sexual violence, with an increased risk of HIV and other Sexually Transmitted Infections (STIs)^6,7^.

Studies conducted in the United States show that transgender individuals who are detained have remarkably high rates of victimization by violence compared to other inmates. According to data from the National Inmate Survey (NIS) in the United States, almost 40% of transgender inmates have been victims of sexual violence, a significant contrast to the rate of only 4% among the other inmates^8^. Another study showed that transgender inmates are thirteen times more likely to suffer sexual assault or rape during their incarceration^9^.

In addition, transgender people face a series of mental health issues, such as low self-esteem, depression, chemical dependence, and suicide attempts, resulting from the adversities to which they are subjected during incarceration^10,11^. During the period of incarceration, this population is confronted with a series of challenges such as restricted access to medical care, scarce preventive measures against infectious diseases, lack of implementation of gender-affirming policies in the prison system, solitary confinement, and prolonged preventive detention^8,10,11^. The incarceration of transgender people is correlated with the decrease in emotional support and social connections after returning to society, resulting in negative impacts on access to the formal labor market or other forms of socioeconomic inclusion^12^. In view of these challenging situations, this population usually resorts to psychoactive substances, which in turn entails other health risks^6,7,9^.

Brazil ranks third globally in terms of prison population, with more than 800 thousand people deprived of freedom^13^. Most inmates in the country are men (94.5%), aged up to 34 years (55%), who identify as Black (54.5%), and with lower levels of education^14^. The Brazilian prison system was highlighted as particularly worrisome in the 2014 United Nations (UN) report, emphasizing the vulnerability of the LGBTI+ population^15^. Furthermore, Brazil leads the global statistics of homicides motivated by sexual orientation and gender identity, intensifying the risks faced by the transgender population^16^.

The investigation of the experience of incarceration among Brazilian *transgend*er women and *travestis* is crucial for understanding the intricate vulnerability factors that permeate this reality. In Latin America, the term “*travesti*” is often adopted by transgender women who face a history marked by extreme vulnerability, in which prostitution assumes the predominant role as a source of livelihood^17^. In these cases, the use of the male reproductive organ to ensure their survival distances these women from the conventional medical concept of “transgender”^18^. In order to preserve the political contextualization and not disregard the history of social and economic marginalization of *travestis*, in the present study we will use the term “*transgend*er women and *travestis*” (TGW) to refer to the group under analysis.

The concept of vulnerability, derived from studies on HIV/AIDS^19^, encompasses a range of individual and collective aspects related to the degree and mode of exposure to a certain disease or condition and, consequently, to greater or lesser access to adequate resources to protect oneself from the undesirable consequences of these situations^20^. This approach focuses on interrelated components: individual vulnerability, which is associated with physical, mental, or behavioral factors increasing the risk of a disease or condition; social vulnerability, which examines cultural and economic dimensions and institutional factors that may determine exposure to diseases, injuries or health conditions; and programmatic vulnerability, which investigates how policies and programs interfere in social and individual conditions^19,20^.

Identifying violent victimization, particularly sexual violence, as well as other risk issues during the period of incarceration, is essential to assist the health system that will welcome this population segment after incarceration so that it is prepared to ensure priority care and safe and humane care strategies^21^. Despite the increased visibility of the transgender population, studies on incarcerated transgender women are still scarce in the Brazilian and global literature^22^ Thus, in this article, we aim to analyze the experience of incarceration of transgender women and *travestis* in five Brazilian capitals.

## METHODS

The data analyzed in this article derive from the *Estudo de Prevalência da Sífilis e outras Infecções Sexualmente Transmissíveis entre Travestis e Mulheres Transexuais no Brasil: Cuidado e Prevenção* (TransOdara)^23^ [“Study on the Prevalence of Syphilis and other Sexually Transmitted Infections among *Travestis* and Transgender Women in Brazil: Care and Prevention”], carried out in five Brazilian capitals (São Paulo, Porto Alegre, Campo Grande, Salvador, and Manaus). We recruited the participants using the Respondent-Driven Sampling (RDS) model^24^. We identified and invited the first participants after an initial formative and exploratory stage; in turn, these participants recruited up to six other transgender women and *travestis* for the research.

The eligibility criteria for the study were to identify as a *travesti*, woman, transgender woman, or other self-designation that implies a female transgender identity not consonant with the male sex assigned at birth; to be 18 years of age or older; to live, work, or study in the cities of the study; and to have a referral coupon provided by a known recruiter, based on the RDS technique. The exclusion criteria were to be under the influence of alcohol or a psychoactive substance in such a way as to make the interview unfeasible.

With the aid of trained researchers, all participants answered a questionnaire containing questions about sociodemographic factors, sexual behavior, drug and alcohol abuse, and experience of violence and incarceration. In addition, the interviewees underwent rapid tests for HIV, Syphilis, Hepatitis B and C, and provided samples of blood, urine, and secretions for further laboratory tests. The project was approved by the Research Ethics Committee of the Santa Casa de Misericórdia de São Paulo (CAAE 05585518.7.0000.5479; opinion n°: 3.126.815 – 30/01/2019), as well as by other participating institutions^23^. All participants signed an Informed Consent Form to participate in the study.

### Variables

The outcome of the present study is the experience of incarceration in life. The question used to construct the outcome was as follows: “Have you ever been arrested in your life?” Initially, a comparison was made between incarcerated and non-incarcerated TGW in order to evaluate possible variables associated with the outcome.

Seeking to understand the experience during the prison period, for participants who reported the experience of incarceration, the reason for the most recent arrest was investigated (open-ended question, subsequently categorized), with whom the participant was incarcerated (only with men; only with women; with other *travestis* and transgender people; or in a specific ward for the LGBTI+ population); and experiences of violence (physical and sexual) suffered in prison. Transgender women and *travestis* who reported spending only one night in prison (42 participants) were not considered in the analyses. The experience of one night detained in a police station is characterized by the penal code as preventive detention or “confinement to prison,” a situation that presupposes evidence of authorship of the crime, but which awaits evidence (Code of Criminal Procedure – Decree-Law 3689/41). In this case, prison confinement is not synonymous with incarceration and would not be sufficient to constitute exposure to the risks explored in this study as a result of the experience of incarceration.

In the analyses, sociodemographic and behavioral variables were also used. The sociodemographic variables were: age group (18 to 24, 25 to 32, 33 to 42, and 43 years or older); self-reported ethnicity/skin color (White, Black, and Asian/Indigenous); level of education (some elementary school; elementary school/some high school; high school/some college); housing situation (own house, rented house, temporarily living with relatives or friends, or homeless); marital status (married or living with a partner; dating or casual dating; separated, widow or single), and whether or not the interviewee is a sex worker. The income variable was categorized based on the minimum wage in force at the time of the study (BRL 1,045.00, about USD 217.15). The main source of income was categorized as sporadic work/without a formal employment contract (also reported as “odd jobs”), sex worker, unemployed, retired or housewife, and work with a formal employment contract. The behavioral variables used were illicit drug use in the last 12 months and information on the first sexual intercourse (whether consensual or forced). Data on verbal, physical, and sexual violence suffered in the 12 months prior to the study were also considered.

### Statistical analysis

The analyses were performed using the Statistical Package for Social Sciences (SPSS, version 22.0). The sample was described by N of the category and percentage. The comparison between the groups was made using Pearson’s χ² homogeneity test. Variables with a p-value of less than or equal to 5% were considered statistically significant.

## RESULTS

A total of 1,245 TGW were included in this study. Among them, the experience of incarceration was reported by 20% (n=253). Those who reported spending only one night at the police station (3.3%, n=42) did not have their experience considered as being an incarceration experience. In Table 1 we present sociodemographic and behavioral data on the TGW, stratified according to the experience of incarceration.

**Table 1:** Sociodemographic and behavioral characteristics of transgender women and *travestis* interviewed about the experience of incarceration.

Variables	Incarceration*		p-value^†^
	Yes (n=253; 20.3)	No (n=992; 79.7)	
Age group (years)			
18 to 24	30 (11.9)	278 (28)	<0.001
25 to 32	85 (33.6)	333 (33.5)	
33 to 42	90 (35.6)	231 (23.2)	
43 or over	48 (19)	152 (15.3)	
Ethnicity/skin color			
White	56 (22.5)	266 (26.9)	0.349
Black	183 (73.5)	688 (69.6)	
Asian/Indigenous	10 (4)	34 (3.4)	
Level of education			
Some elementary school	115 (45.5)	188 (19)	<0.001
Elementary school/some high school	115 (45.5)	566 (57.1)	
High school/some college/university degree	23 (9.1)	237 (23.9)	
Income (in minimum wage)			
Up to 1 salary	106 (46.9)	453 (49.8)	0.481
1 to 2 salaries	89 (39.4)	319 (35.1)	
2 or more salaries	31 (13.7)	137 (15.1)	
Main source of income			
Sporadic work/without a formal employment contract	76 (30.3)	366 (37)	<0.001
Sex worker	75 (29.9)	190 (19.2)	
Unemployed	49 (19.5)	236 (23.8)	
Retired/housewife	34 (13.5)	92 (9.3)	
Formal employment contract	17 (6.8)	106 (10.7)	
Housing situation			
Own house	49 (21.4)	273 (28)	<0.001
Rented house	95 (41.5)	368 (37.8)	
Temporarily living with relatives/friends	52 (22.7)	272 (27.9)	
Homeless	33 (14.4)	61 (6.3)	
Marital status			
Married/living together	49 (19.4)	129 (13)	0.035
Dating/Casual dating	34 (13.4)	139 (14)	
Separated/widow/single	170 (67.2)	724 (73)	
Sex worker			
Yes	75 (29.6)	190 (19.1)	<0.001
No	207 (70.4)	804 (80.9)	
History of illicit drug use			
Yes	168 (66.4)	507 (51)	<0.001
No	85 (33.8)	487 (49)	

* Totals may differ due to incompleteness of data;

† Equality of proportions test based on Pearson’s chi-square statistics.

The experience of incarceration was reported more frequently among TGW aged between 33 and 42 years (35.6%, p<0.001), who identify as Black (73.5%), with lower level of education (p<0.001), living in a rented house (p<0.001), and without a partner (p=0.035). There was a predominance of incarcerated TGW who had a history of drug use in the 12 months prior to the study (66.4%, p<0.001). About 30% of transgender women and *travestis* with a history of incarceration were sex workers at the time of the study.

In Table 2 we show the experience of violence suffered by TGW during life and in the last year, stratified by the experience of incarceration. In the last 12 months, 48.8% of incarcerated transgender women and *travestis* reported having been victims of verbal violence, and 24.2% of physical violence. Lifetime sexual violence was prevalent in the whole sample, and it was 52.6% among incarcerated TGW. Having suffered physical violence in the last 12 months was associated with the experience of incarceration among the interviewed TGW (p<0.001).

**Table 2:** Characteristics of transgender women and *travestis* interviewed about violence suffered in the last 12 months and throughout life.

Variables	Incarceration*		p-value^†^
	Yes (n=253; 20.3)	No (n=992; 79.7)	
First sexual intercourse			
Consented	219 (86.9)	815 (84.6)	0.367
Forced	33 (13.1)	148 (15.4)	
Verbal violence (12 months)			
Yes	122 (48.8)	457 (46.4)	0.496
No	128 (51.2)	528 (53.6)	
Physical violence (12 months)			
Yes	61 (24.2)	130 (13.2)	<0.001
No	191 (75.8)	857 (86.8)	
Sexual violence (life)			
Yes	133 (52.6)	498 (50.5)	0.549
No	120 (47.4)	489 (49.5)	

* Totals may differ due to incompleteness of data;

† Equality of proportions test based on Pearson’s chi-square statistics.

In Table 3 we present the experiences during incarceration. Most of the TGW reported having been imprisoned with cisgender men (60.9%). Just over 30% of the TGW reported having stayed in a specific ward with other transgender women or *travestis*, and 4.7% of them reported having been arrested along with other cisgender women.

**Table 3:** Incarceration experience of transgender women and *travestis* concerning the place of incarceration, reason for imprisonment, and violence suffered in prison.

Variable	Incarceration (n=253)*	%
Ward Type		
Stayed in LGBTI+ ward	10	4
Stayed with other men	154	60.9
Stayed with other women	12	4.7
Stayed with other TGW	77	30.4
Reason for arrest		
Robbery	70	29.2
Drug trafficking	73	30.4
Prostitution	21	8.8
Fighting/assault	17	7.1
Homicide	21	8.8
Others^†^	38	15.7
Has been assaulted in prison		
Yes	66	26.3
No	185	73.7
Has suffered sexual violence in prison		
Yes	35	13.8
No	218	86.2

TGW: transgender women and *travestis*.

* Excluding those who spent one night in prison (n=42);

† The “Others” category includes: “being in the wrong place/wrong time/going along” (n=9), fraud (n=4), contempt (n=5), disorder (n=5), pimping (n=4), illegal immigration (n=4), damage to public property (n=1), carrying a weapon (n=2), criminal misrepresentation (n=2), and pedophilia (n=2).

The most frequent reasons for arrest were drug trafficking (30.4%) and robbery (29.2%). Although sex work is not a crime in Brazil, 8.8% of TGW reported having been arrested for prostitution. The reason why the TGW were incarcerated was an open-ended question (and not an analysis of their criminal cases). Therefore, other reasons emerged in the study such as “being in the wrong place/wrong time.”

The experience of violence within prison was frequent among the TGW. More than a quarter of the interviewees were assaulted during incarceration (26.3%) and 13.8% reported having suffered sexual violence during incarceration.

## DISCUSSION

The present study aimed to evaluate the experience of incarceration among TGW in Brazil. The results showed that 20.3% of the analyzed sample (n=253) had experienced being deprived of freedom at least once in their lives. The prevalence of incarceration observed in our study is similar to that reported in other investigations, ranging from 19.3^11^ to 21%^4^. Among the TGW who participated in this research and most frequently reported the experience of incarceration, those aged between 33 and 42 years, with a lower level of education, absence of regular occupation, history of illicit drug use, and report of physical violence in the last 12 months stand out.

According to the current literature, several individual risk factors are correlated with the experience of incarceration among TGW, including low level of education, low socioeconomic status, engagement in sex work, and homelessness^4,11,25^. Sex work emerges as a significant possible predictor of incarceration for TGW, as more than one-fifth of the study participants had sex work as their main source of income, and of these, almost 30% had been detained at some point in their lives. As pointed out by Hughto et al., 27% of incarcerated transgender women were or had been sex workers, being 2.6 times more likely to have engaged in sex work compared to non-incarcerated women^26^. The higher incidence of incarceration among TGW who were homeless, as well as those who temporarily lived with relatives or friends, also finds parallels in this study, in which almost half (48%) of incarcerated transgender women had already experienced homelessness^26^.

In addition to these individual characteristics, there are social factors, such as discrimination concerning gender identity, which may have implications for higher school dropouts and difficulty in accessing the labor market^1,26^. The structural stigma associated with transgenderism^27^ can also affect TGW in the treatment they receive from law enforcement and the justice system, placing them in a situation of vulnerability to the experience of incarceration.

In Brazil, as in other countries, transgender women who have not undergone sex reassignment surgeries are placed in male facilities^4,7^, where they are potentially exposed to a higher risk of verbal, physical, and sexual assault^1,28^. In this study, a considerable part of the sample (60.9%) of TGW was allocated to male prisons. According to Jeness and Sexton, men’s prisons are environments of expanded policing for sexual and gender nonconformities. Individuals who transgress gender norms are placed lower in the hierarchy of social status, are less respected, and become extremely vulnerable to violence^9^. As ways of dealing with this violence, transgender women end up “voluntarily” becoming sexually involved with other prisoners to avoid being assaulted^29^. The qualitative study by Oliveira et al., conducted in Brazil, also shows that the incarceration of *travestis* in male wards amplifies other forms of violence intrinsic to the prison system, such as rebellions, in which *travestis*, along with sexual abusers, are considered the main targets of inmates^30^.

To meet the specificities of the LGBTI+ population deprived of freedom, the Council of Justice published Resolution No. 348, of 10/13/2020, which regulates the guidelines and procedures to be adopted by the Judicial Branch in the treatment of LGBTI+ people deprived of freedom^31^. The document assures *transgend*er women and *travestis* the same rights reserved for cisgender women, as well as the judge’s obligation to inquire about the preference for incarceration in a female or male unit or, in cases where available, in a specific unit for the LGBTI+ population. Despite a great advance in terms of dignified treatment of the population of TGW, this resolution still faces major obstacles to its implementation: lack of training of professionals in the judicial and prison system, lack of specific wards, precariousness of health care adequate to the specificities of this group in prisons, among others^32^.

In the present study, drug trafficking was the main reason for the incarceration of TGW. This datum is similar to the situation of cisgender women. According to data from the Brazilian Ministry of Justice and Public Security, the female prison population is marked by convictions for drug crimes (drug trafficking and association for trafficking, Law 6.368/76 and Law 11.343/06), with 55.4% of women being incarcerated in this group of crimes, while 27.5% of men are in the same situation^32^. In Brazil, female imprisonment is combined with indicators of social vulnerability of these women such as unemployment, low level of education, and history of drug abuse^33^. Among the current explanations for the greater participation of cisgender women in drug trafficking is their relationship with intimate partners or family members, who are already involved in drug trafficking^31^. There are also studies that evidence a personal choice, in which the woman would have the opportunity to gain greater recognition and social status in her community^34,35^.

For *transgend*er women and *travestis*, however, there is little research on the topic. The involvement of TGW with drug trafficking may be associated with a higher prevalence of illicit drug use by this population^2,3^. As sex work is not considered a crime in the country, the mere possession of drugs by TGW sex workers can be used as a reason for their arrest. In addition, their involvement with drug trafficking may be due to factors such as discrimination and prejudice which culminate in a lack of formal work opportunities, in such a way that drug trafficking may be perceived by them as a potential source of income. A qualitative study conducted with transgender women imprisoned in Nicaragua and El Salvador demonstrated that the lack of formal employment opportunities was the main reported reason for them to commit crimes^29^. As in our results, the reasons for imprisonment cited in the study were drug trafficking, robbery, and prostitution^29^.

The profile of TGW with experiences of incarceration evidences the situation of greater social vulnerability of these transgender women and *travestis*. The stigma associated with transgenderism^27^ can have direct implications on living conditions, such as low level of education, difficult insertion in the labor market, sex work, and homelessness, making incarceration a frequent situation. Despite the legal advances in the treatment of the LGBTI+ population deprived of freedom, which grants TGW the same rights reserved for cisgender women, in this article we demonstrate that the percentage of TGW with violation of rights was high, and the difficulties in effectively guaranteeing the rights of this population are evidenced.

Several forms of violence, in addition to those occurring in the prison environment, showed high prevalence in the studied sample. These data reflect the high incidence of gender-based violence (GBV) faced by TGW in different contexts^36^. GBV is an umbrella term that encompasses any form of physical, sexual, or emotional violence perpetrated against a person’s will and stemming from power inequalities based on gender roles^37^. As highlighted by Wirtz et al., the failure of the State to recognize the identity of transgender people and to protect them against discrimination contributes to the creation of environments conducive to the occurrence of violence, which is sometimes tolerated or even normalized due to the absence of adequate sanctions^36^.

The fact that the incarceration of TGW takes place in female or male units or specific units for the LGBTI+ population may be related to the various situations of violence experienced in prison. Despite the robust evidence that the allocation of TGW in male wards is detrimental to ensuring the physical and psychological well-being of these inmates^4,6,7,9^, we emphasize the need for studies that analyze the experience of TGW incarceration with cisgender women or in specific wards for the LGBTI+ population.

To ensure the rights of transgender women and *travestis* deprived of freedom in Brazil, it is essential to implement government initiatives in line with the guidelines and norms recommended by the resolution of the Council of Justice. In addition, public policy initiatives are necessary to mitigate the contexts of vulnerability that affect this population. These measures include the implementation of quota policies in higher education, the adoption of affirmative policies in the labor market to expand employment opportunities, the creation of housing policies and, finally, the development of policies that promote greater gender equity. These collective actions are crucial to promote the inclusion, protection, and guarantee of the fundamental rights of this community.

It is worth highlighting some limitations inherent in the present study. The main one is related to the difficulty in establishing the temporal relationship between incarceration and other situations experienced by the interviewees such as the sociodemographic status. In addition, considering that the main focus of the research was not incarceration, few questions on this specific topic were included in the questionnaire. The choice of the RDS technique, due to the nature of the population that is difficult to access, results in convenience sampling. Furthermore, the study was conducted in five Brazilian state capitals, which do not cover all states or TGW living in rural areas. Consequently, the results should be interpreted with caution and cannot be generalized to the entire population of *transgend*er women and *travestis*. Considering the need for a more comprehensive understanding, other studies investigating diverse experiences of TGW during incarceration, especially in Latin American countries, would be valuable to broaden the understanding of this panorama.
