# Erratum

**DOI:** 10.1590/1980-549720240014.supl.1erratum

**Published:** 2024-09-30

In the manuscript "**Profile and experiences during the incarceration of transgender women and travestis (TGW) in Brazil: a cross-sectional study**", DOI: https://doi.org/10.1590/1980-549720240014.supl.1, published in the Rev Bras Epidemiol. 2024; 27(Suppl 1): e240014.supl.1:

**Where it reads:**

Maria Amélia de Sousa Mascena Vera

**It should read:**

Maria Amélia de Sousa Mascena Veras

