# Supplementary material for: Profile and experiences during the incarceration of transgender women and travestis (TGW) in Brazil: a cross-sectional study
Source: Rev Bras Epidemiol. 2024 Aug 19;27(Suppl 1):e240014.supl.1. doi: 10.1590/1980-549720240014.supl.1 (PMC11338535; doi:10.1590/1980-549720240014.supl.1)
Supplement: Translation

# Translation: Perfil e experiências durante o encarceramento de mulheres trans e travestis (MTT) no Brasil: um estudo transversal

**DOI:** 10.1590/1980-549720240014.supl.1.2

**Authors:** Andréa Fachel Leal, Cristine Coelho Cazeiro, Ana Carolina Einsfeld Mattos, Bruna Hentges, Luciana Barcellos Teixeira, Daniela Riva Knauth, Laio Magno, Inês Dourado, Maria Amélia de Sousa Mascena Vera

**Affiliations:** I Universidade Federal do Rio Grande do Sul, Departamento de Sociologia – Porto Alegre (RS), Brasil.; II Universidade Federal do Rio Grande do Sul, Programa de Pós-Graduação em Epidemiologia – Porto Alegre (RS), Brasil.; III Universidade Federal do Rio Grande do Sul, Programa de Pós-Graduação em Políticas Públicas – Porto Alegre (RS), Brasil.; IV Universidade Federal do Rio Grande do Sul, Departamento de Saúde Coletiva – Porto Alegre (RS), Brasil.; V Universidade Federal do Rio Grande do Sul, Departamento de Medicina Social – Porto Alegre (RS), Brasil.; VI Universidade do Estado da Bahia – Salvador (BA), Brasil.; VII Universidade Federal da Bahia – Salvador (BA), Brasil.; VIII Santa Casa de São Paulo, Faculdade de Ciências Médicas – São Paulo (SP), Brasil.

**Keywords:** Pessoas transgênero, Travestilidade, Pessoas privadas de liberdade, Prisão, Vulnerabilidade social

## Abstract

**Objetivo:**

O objetivo do presente estudo é descrever as características sociodemográficas e comportamentais de um grupo de mulheres trans e travestis (MTT) com histórico de encarceramento e o contexto institucional e social desta experiência no Brasil.

**Métodos:**

Os dados são provenientes do Estudo TransOdara, de delineamento transversal, realizado em 5 capitais brasileiras no período de dezembro de 2019 a julho de 2021. As participantes foram recrutadas pela técnica *Respondent-Driven Sampling* (RDS), onde, após uma etapa inicial formativa e exploratória, as primeiras participantes foram identificadas; elas, por sua vez, recrutavam até outras seis mulheres trans e travestis para a pesquisa. O desfecho do estudo foi a experiência de encarceramento durante a vida apreendido através da pergunta: “Você alguma vez na vida já foi presa?”.

**Resultados:**

Um total de 1.245 MTT foram entrevistadas. Destas, 20,3% (n=253) experienciaram o cárcere. O encarceramento foi mais frequente entre as entrevistadas de 33 a 42 anos (35,6%), com menor escolaridade (45,5%), em situação de trabalho informal (30,3%) e entre aquelas que reportaram uso de drogas ilícitas (66,4%). A maioria (60,9%) das MTT ficou presa com homens cisgênero, e o motivo da prisão mais frequente foi o tráfico de drogas (30,4%), seguido de roubo (29,2%). Mais de um quarto das entrevistadas (26,3%) sofreu agressão, e 13,8% relataram ter sofrido violência sexual durante o encarceramento.

**Conclusão:**

Os resultados destacam a elevada prevalência de encarceramento entre MTT. Este encarceramento se dá em alas masculinas e em um contexto de altas taxas de violência física e sexual.

## INTRODUÇÃO

Pessoas trans sofrem diversas formas de discriminação e violências baseadas no gênero, que restringem seu acesso a recursos necessários para o alcance e manutenção de uma vida digna e saudável, como educação, emprego e renda^1,2^. Consequentemente, indivíduos trans frequentemente se veem envolvidos em atividades informais, como o trabalho sexual, ou mesmo em atividades ilícitas, como o tráfico de entorpecentes^3^.

O estigma e a consequente exclusão social das pessoas trans impactam nos índices de encarceramento e na forma como são tratadas no sistema de justiça criminal, fazendo com que as taxas de encarceramento de indivíduos trans sejam maiores do que a população geral^4,5^. A experiência de encarceramento para pessoas trans ocasiona uma maximização das violações sofridas em liberdade: violência física, psicológica e sexual, com risco aumentado de HIV e outras Infecções Sexualmente Transmissíveis (IST’s)^6,7^.

Estudos realizados nos Estados Unidos revelam que indivíduos trans que estão detidos apresentam taxas notavelmente elevadas de vitimização por violência em comparação com outros reclusos. De acordo com dados do *National Inmate Survey (NIS)* dos Estados Unidos, quase 40% dos detentos trans foram vítimas de violência sexual, contrastando significativamente com a taxa de apenas 4% entre os demais presos^8^. Outro estudo demonstrou que detentos trans têm uma probabilidade treze vezes maior de sofrer agressão sexual ou estupro durante o período de encarceramento^9^.

Adicionalmente, pessoas trans enfrentam de maneira exacerbada uma série de problemas de saúde mental, como baixa autoestima, depressão, dependência química e tentativas de suicídio, decorrentes das adversidades a que são submetidas durante o encarceramento^10,11^. No período de reclusão, essa população é confrontada com uma série de desafios, tais como acesso restrito a cuidados médicos, escassas medidas preventivas contra doenças infecciosas, carência na implementação de políticas de afirmação de gênero no sistema prisional, confinamentos solitários e detenções preventivas prolongadas^8,10,11^. O encarceramento de pessoas trans está correlacionado a declínios no suporte emocional e nas conexões sociais após o retorno à sociedade, resultando em impactos negativos no acesso ao mercado de trabalho formal ou outras formas de inserção socioeconômica^12^. Diante dessas situações desafiadoras, é comum o recurso a substâncias psicoativas nessa população, acarretando, por sua vez, outros riscos à saúde^6,7,9^.

O Brasil ocupa a terceira posição global em população carcerária, com mais de 800 mil pessoas privadas de liberdade^13^. A maioria dos detentos no país é composta por homens (94,5%), com idade até 34 anos (55%), pertencentes a grupos étnicos negros que se identificam como negros (54,5%) e com níveis educacionais mais baixos^14^. O sistema prisional brasileiro foi destacado como particularmente preocupante no relatório de 2014 da Organização das Nações Unidas (ONU), enfatizando a vulnerabilidade da população LGBTI+^15^. Além disso, o Brasil lidera as estatísticas globais de homicídios motivados pela orientação sexual e identidade de gênero, intensificando os riscos enfrentados pela população trans^16^.

A investigação da experiência de encarceramento entre as mulheres trans e travestis brasileiras é crucial para a compreensão dos intrincados fatores de vulnerabilidade que permeiam essa realidade. Na América Latina, o termo “travesti” é frequentemente adotado por mulheres trans que enfrentam uma história marcada por extrema vulnerabilidade, em que a prostituição assume o papel predominante como fonte de subsistência^17^. Nestes casos, a utilização do órgão sexual masculino para garantir sua sobrevivência afasta essas mulheres do conceito médico convencional de “transgênero”^18^. Para preservar a contextualização política e não desconsiderar o histórico de marginalização social e econômica das travestis, o presente estudo optará por utilizar o termo “mulheres trans e travestis” (MTT) para referir-se ao grupo em análise.

O conceito de vulnerabilidade, oriundo de estudos sobre HIV/Aids^19^, abrange uma gama de aspectos individuais e coletivos relacionados ao grau e modo de exposição a determinadas situações e, consequentemente, ao maior ou menor acesso a recursos adequados para proteger-se das consequências indesejáveis dessas situações^20^. Essa abordagem analisa componentes inter-relacionados: a vulnerabilidade individual, que está associada a fatores físicos, mentais ou comportamentais aumentando o risco de doença; a vulnerabilidade social, que examina dimensões culturais, econômicas e fatores institucionais que podem determinar a exposição a doenças ou agravos; e a vulnerabilidade programática, que investiga como políticas e programas interferem em situações sociais e individuais^19,20^.

Identificar vitimização violenta, particularmente a violência sexual, bem como outras questões de risco durante o período de encarceramento, é fundamental para auxiliar o sistema de saúde que irá acolher esse segmento populacional após o cárcere, para que esteja preparado para garantir atenção prioritária e estratégias seguras e humanas de cuidado^21^. Apesar do aumento da visibilidade da população trans, estudos sobre mulheres trans encarceradas ainda são escassos na literatura brasileira e mundial^22^. Desta forma, o presente artigo tem por objetivo analisar a experiência de encarceramento de mulheres trans e travestis em cinco capitais brasileiras.

## MÉTODOS

Os dados analisados neste artigo são provenientes do Estudo de Prevalência da Sífilis e outras Infecções Sexualmente Transmissíveis entre Travestis e Mulheres Transexuais no Brasil: Cuidado e Prevenção (TransOdara)^23^, realizado em cinco capitais brasileiras (São Paulo, Porto Alegre, Campo Grande, Salvador e Manaus). As participantes foram recrutadas pelo modelo *Respondent-Driven Sampling (RDS)* ^24^. Após uma etapa inicial formativa e exploratória, as primeiras participantes foram identificadas; elas, por sua vez, recrutavam até outras seis mulheres trans e travestis para a pesquisa.

Os critérios de elegibilidade para o estudo eram identificar-se como travesti, mulher, mulher trans ou outra autodesignação que implique uma identidade de gênero trans feminina não consonante ao sexo masculino atribuído ao nascimento; ter 18 anos ou mais; residir, trabalhar ou estudar nas cidades do estudo; e possuir cupom de encaminhamento fornecido por um recrutador (a) conhecido (a), a partir da técnica de amostragem RDS. Os critérios de exclusão foram encontrar-se sob efeito de álcool ou substância psicoativa de tal modo que impossibilitasse a entrevista.

Com a ajuda de pesquisadores treinados, todas as participantes responderam a um questionário contendo questões sociodemográficas, de comportamento sexual, abuso de drogas e álcool, e experiência de violência e encarceramento. Além disso, as entrevistadas realizaram testes rápidos para HIV, Sífilis, Hepatite B e C, e forneceram amostras de sangue, urina e secreções para testes laboratoriais posteriores. O projeto foi aprovado pelo Comitê de Ética em Pesquisa (CEP) da Santa Casa de Misericórdia de São Paulo (CAAE 05585518.7.0000.5479 - Nº parecer: 3.126.815 - 30/01/2019), assim como pelas demais instituições participantes^23^. Todas as participantes assinaram um Termo de Consentimento Livre e Esclarecido para participar do estudo.

### Variáveis

O desfecho do presente estudo é a experiência de encarceramento na vida. A pergunta utilizada para construção do desfecho foi a seguinte: “Você alguma vez na vida já foi presa?”. Inicialmente, é feita uma comparação entre MTT encarceradas e não encarceradas, a fim de avaliarmos possíveis variáveis associadas ao desfecho.

Buscando entender a experiência durante o período prisional, para as participantes que relataram a experiência de cárcere, foi investigado a razão da prisão mais recente (pergunta aberta, categorizada posteriormente), com quem a participante ficou encarcerada (apenas com homens; apenas com mulheres; com outras mulheres trans e travestis; ou em uma ala específica para população LGBTI+); e experiências de violências (física e sexual) sofridas na prisão. Não foram consideradas nas análises as mulheres trans e travestis que relataram ter passado apenas uma noite na prisão (42 participantes). A experiência de uma noite detida em delegacia é caracterizada pelo código penal como prisão preventiva ou “recolhimento à prisão”, situação que pressupõe indícios de autoria do crime, mas que aguarda provas (Código Processo Penal – Decreto-lei 3689/41). Neste caso, o recolhimento à prisão não é sinônimo de encarceramento, e não seria suficiente para configurar exposição aos riscos explorados neste estudo em decorrência da experiência de cárcere.

Nas análises, variáveis sociodemográficas e comportamentais também foram utilizadas. As variáveis sociodemográficas foram: faixa etária (18 a 24, 25 a 32, 33 a 42 e 43 anos ou mais); raça/cor autodeclarada (branca, negra e amarela/indígena); escolaridade (até ensino fundamental incompleto; ensino fundamental completo/ensino médio incompleto; ensino médio completo/superior incompleto); situação de moradia (casa própria, casa alugada, vivendo provisoriamente com parentes ou amigos, ou em situação de rua); situação conjugal (casada ou morando junto com parceiro(a); namorando ou ficando; separada, viúva ou solteira) e se a entrevistada é ou não profissional do sexo. A variável renda foi categorizada a partir do salário mínimo vigente no momento do estudo (R$ 1.045,00, cerca de $217.15 dólares). A principal fonte de renda foi categorizada em: trabalho esporádico/sem carteira assinada (também relatado como “bicos”), profissional do sexo, desempregada, aposentada ou dona de casa e trabalho com carteira assinada. As variáveis comportamentais utilizadas foram uso de drogas ilícitas nos últimos 12 meses e informações sobre a primeira relação sexual (se consentida ou forçada). Foram utilizados também dados sobre violência verbal, física e sexual sofrida nos 12 meses anteriores ao estudo.

### Análise estatística

As análises foram realizadas utilizando o *software Statistical Package for Social Sciences* (SPSS, versão 22.0). A amostra foi descrita por N da categoria e percentual. A comparação entre os grupos utilizou o teste de homogeneidade do χ² de Pearson. Foram considerados estatisticamente significativas as variáveis que apresentaram valor de p menor ou igual a 5%.

## RESULTADOS

Um total de 1.245 MTT foram incluídas neste estudo. Dentre essas, a experiência de encarceramento foi reportada por 20% (n=253) delas. Não se considerou como experiência de encarceramento aquela reportada por 3,3% (n=42) da amostra, que referiram passar apenas uma noite na delegacia. A Tabela 1 apresenta dados sociodemográficos e comportamentais das MTT, estratificadas de acordo com a experiência de encarceramento.

**Tabela 1:** Características sociodemográficas e comportamentais de mulheres trans e travestis entrevistadas em relação à experiência de encarceramento.

Variáveis	Encarceramento*		Valor de p^†^
	Sim (n=253; 20,3)	Não (n=992; 79,7)	
Faixa etária (anos)			
18 a 24	30 (11,9)	278 (28)	<0.001
25 a 32	85 (33,6)	333 (33,5)	
33 a 42	90 (35,6)	231 (23,2)	
43 ou mais	48 (19)	152 (15,3)	
Raça/cor			
Branca	56 (22,5)	266 (26,9)	0.349
Negra	183 (73,5)	688 (69,6)	
Amarela/indígena	10 (4)	34 (3,4)	
Escolaridade			
Ensino fundamental incompleto	115 (45,5)	188 (19)	<0.001
Ensino fundamental completo/ensino médio incompleto	115 (45,5)	566 (57,1)	
Ensino médio completo/superior incompleto	23 (9,1)	237 (23,9)	
Renda (em salário mínimo)			
Até 1	106 (46,9)	453 (49,8)	0.481
1 a 2	89 (39,4)	319 (35,1)	
2 ou mais	31 (13,7)	137 (15,1)	
Principal fonte de renda			
Trabalho esporádico/sem carteira assinada	76 (30,3)	366 (37)	<0.001
Profissional do sexo	75 (29,9)	190 (19,2)	
Desempregada	49 (19,5)	236 (23,8)	
Aposentada/dona de casa	34 (13,5)	92 (9,3)	
Carteira assinada	17 (6,8)	106 (10,7)	
Situação de moradia			
Casa própria	49 (21,4)	273 (28)	<0.001
Casa alugada	95 (41,5)	368 (37,8)	
Provisório com parentes/amigos	52 (22,7)	272 (27,9)	
Em situação de rua	33 (14,4)	61 (6,3)	
Situação conjugal			
Casada/morando junto	49 (19,4)	129 (13)	0.035
Namorando/ficando	34 (13,4)	139 (14)	
Separada/viúva/solteira	170 (67,2)	724 (73)	
Profissional do sexo			
Sim	75 (29,6)	190 (19,1)	<0.001
Não	207 (70,4)	804 (80,9)	
Histórico de uso de drogas ilícitas			
Sim	168 (66,4)	507 (51)	<0.001
Não	85 (33,8)	487 (49)	

* Totais podem diferir por incompletude dos dados;

† Teste de igualdade de proporções baseado na estatística qui-quadrado de Pearson.

A experiência de encarceramento foi reportada mais frequentemente entre MTT com idade entre 33 a 42 anos (35,6%, p<0.001), de raça/cor autodeclarada preta ou parda (negra) (73,5%), com menor escolaridade (p<0.001), que vivem em uma casa alugada (p<0.001) e sem parceiro(a) (p=0.035). Houve um predomínio de MTT encarceradas que possuíam histórico de uso de drogas nos 12 meses anteriores ao estudo (66,4%, p<0.001). Cerca de 30% das mulheres trans e travestis com histórico de encarceramento eram profissionais do sexo no momento do estudo.

A Tabela 2 demonstra a experiência de violência sofridas por MTT durante a vida e no último ano, estratificadas pela experiência de encarceramento. Nos últimos 12 meses, 48,8% das mulheres trans e travestis encarceradas reportaram ter sido vítimas de violência verbal, e 24,2% de violência física. A violência sexual durante a vida foi prevalente em toda a amostra, e entre as MTT encarceradas foi de 52,6%. Ter sofrido violência física nos últimos 12 meses esteve associada à experiência de encarceramento entre as MTT entrevistadas (p<0.001).

**Tabela 2:** Características de mulheres trans e travestis entrevistadas em relação a violências sofridas nos últimos 12 meses e ao longo da vida.

Variáveis	Encarceramento*		Valor de p^†^
	Sim (n=253; 20,3)	Não (n=992; 79,7)	
Primeira relação sexual			
Consentida	219 (86,9)	815 (84,6)	0.367
Forçada	33 (13,1)	148 (15,4)	
Violência verbal (12 meses)			
Sim	122 (48,8)	457 (46,4)	0.496
Não	128 (51,2)	528 (53,6)	
Violência física (12 meses)			
Sim	61 (24,2)	130 (13,2)	<0.001
Não	191 (75,8)	857 (86,8)	
Violência sexual (vida)			
Sim	133 (52,6)	498 (50,5)	0.549
Não	120 (47,4)	489 (49,5)	

* Totais podem diferir por incompletude dos dados;

† Teste de igualdade de proporções baseado na estatística qui-quadrado de Pearson

As experiências durante o cárcere são apresentadas na Tabela 3. A maior parte das MTT afirmou ter ficado presa com homens cisgênero (60,9%). Pouco mais de 30% das MTT afirmou ter ficado em uma ala específica com outras mulheres trans e travestis, e 4,7% delas reportaram ter sido presas junto com outras mulheres cisgênero.

**Tabela 3:** Experiência de encarceramento em mulheres trans e travestis em relação ao local de privação, motivo para o cárcere e violência sofrida no cárcere.

Variável	Encarceramento (n=253)*	%
Tipo de ala		
Ficou em ala LGBTI+	10	4
Ficou com outros homens	154	60,9
Ficou com outras mulheres	12	4,7
Ficou com outras MTT	77	30,4
Motivo da prisão		
Roubo	70	29,2
Tráfico de drogas	73	30,4
Prostituição	21	8,8
Briga/agressão	17	7,1
Homicídio	21	8,8
Outros^†^	38	15,7
Sofreu agressão na prisão		
Sim	66	26,3
Não	185	73,7
Sofreu violência sexual na prisão		
Sim	35	13,8
Não	218	86,2

MTT: mulheres trans e travestis.

* Excluindo as que passaram uma noite na prisão (n=42);

† A categoria outros inclui: “estar no lugar errado/hora errada/ir junto” (n=9), estelionato (n=4), desacato (n=5), desordem (n=5), cafetinagem (n=4), imigração ilegal (n=4), dano ao patrimônio público (n=1), porte de arma (n=2), falsidade ideológica (n=2) e pedofilia (n=2).

Os motivos mais frequentes para prisão foram tráfico de drogas (30,4%) e roubo (29,2%). Embora o trabalho sexual não seja crime no Brasil, 8,8% das MTT relataram ter sido presas por prostituição. A razão pela qual as MTT foram encarceradas se tratava de uma pergunta aberta (e não de uma análise de seus processos penais). Por isso, outros motivos como este surgiram no estudo, como “estar no lugar errado/hora errada”.

A experiência de violência dentro da prisão se mostrou frequente entre as MTT. Mais de um quarto das entrevistadas sofreu agressão durante o cárcere (26,3%) e 13,8% relataram ter sofrido violência sexual durante o encarceramento.

## DISCUSSÃO

O presente estudo teve como objetivo avaliar a vivência de encarceramento entre as MTT no Brasil. Os resultados revelaram que 20,3% da amostra analisada (n=253) experimentou pelo menos uma privação de liberdade ao longo da vida. A prevalência de encarceramento observada em nosso estudo assemelha-se àquela reportada em outras investigações, variando de 19,3^11^ a 21%^4^. Entre as MTT que participaram desta pesquisa e relataram mais frequentemente a experiência de encarceramento, destacam-se aquelas com idades entre 33 e 42 anos, menor nível educacional, ausência de ocupação regular, histórico de uso de drogas ilícitas e relato de violência física nos últimos 12 meses.

Conforme a literatura atual, diversos fatores de risco individuais estão correlacionados à vivência de encarceramento entre as MTT, incluindo baixo nível educacional, baixo *status* socioeconômico, envolvimento em trabalho sexual e falta de moradia^4,11,25^. O trabalho sexual emerge como um possível preditor significativo de encarceramento para as MTT, uma vez que mais de um quinto das participantes do estudo tinham no trabalho sexual sua principal fonte de renda, e destas, quase 30% haviam sido detidas em algum momento de suas vidas. Conforme apontado por Hughto et al., 27% das mulheres trans encarceradas eram ou haviam sido profissionais do sexo, apresentando 2,6 vezes mais probabilidade de terem se envolvido em trabalho sexual em comparação com aquelas não encarceradas^26^. A maior incidência de encarceramento entre as MTT que viviam em situação de rua, assim como aquelas que residiam temporariamente na casa de parentes ou amigos, também encontra paralelos neste estudo, onde quase metade (48%) das mulheres trans encarceradas já havia experimentado a falta de moradia ou abrigo^26^.

Agrega-se a essas características individuais os fatores sociais, como a discriminação em relação à identidade de gênero, que pode ter implicações sobre maior evasão escolar e dificuldade de acesso ao mercado de trabalho^1,26^. O estigma estrutural, associado à transgeneridade^27^, também pode afetar as MTT no tratamento que recebem dos operadores de Direito e do sistema de justiça, colocando-as em situação de vulnerabilidade para a própria experiência da privação de liberdade.

No Brasil, assim como em outros países, as mulheres trans que não passaram por cirurgia de redesignação sexual são colocadas em instalações masculinas^4,7^, onde estão potencialmente expostas a um maior risco de agressão verbal, física e sexual^1,28^. Neste estudo, grande parte da amostra (60,9%) de MTT ficou alocada em prisões masculinas. Segundo Jeness e Sexton, prisões masculinas são ambientes de policiamento ampliado para inconformidades sexuais e de gênero. Aqueles que transgridem as normas de gênero são situados em posição inferior na hierarquia do estatuto social, gozam de menos respeito e tornam-se extremamente vulneráveis à violência^9^. Como formas de lidar com essas violências, mulheres trans acabam por se envolver sexualmente de forma “voluntária” com outros presos, para evitar agressões^29^. O estudo qualitativo de Oliveira et al., realizado no Brasil, também evidencia que o encarceramento de travestis em alas masculinas amplifica outras violências intrínsecas ao sistema prisional, como em rebeliões, onde as travestis, juntamente com os abusadores sexuais, são consideradas os principais alvos dos detentos^30^.

Visando atender as especificidades da população LGBTI+ privada de liberdade, o Conselho de Justiça publicou a Resolução n^o^ 348, de 13/10/2020, que normatiza as diretrizes e os procedimentos a serem adotadas pelo Poder Judiciário no tratamento de pessoas LGBTI+ privadas de liberdade^31^. O documento assegura às mulheres trans e travestis os mesmos direitos reservados às mulheres cisgêneros, bem como a obrigação do juiz de indagar acerca da preferência de encarceramento em unidade feminina, masculina ou, nos casos onde houver disponibilidade, em unidade específica para população LGBTI+. Apesar de um grande avanço em termos do tratamento digno à população de MTT, tal resolução ainda encontra grandes obstáculos para sua implementação: falta de capacitação dos profissionais do sistema judiciário e prisional, falta de alas específicas, precariedade do atendimento em saúde adequado às especificidades deste grupo nas prisões, entre outras^32^.

No presente estudo, o tráfico de drogas foi o principal motivo para o encarceramento de MTT. Esse dado se assemelha ao que ocorre com mulheres cisgênero. Segundo dados do Ministério da Justiça e Segurança Pública, a população prisional feminina é marcada por condenações por crimes de drogas (tráfico de drogas e associação para o tráfico, Lei 6.368/76 e Lei 11.343/06), sendo que 55,4% das mulheres estão presas neste grupo de crimes, enquanto 27,5% dos homens se encontram na mesma situação^32^. No Brasil, o aprisionamento feminino está combinado a indicadores de vulnerabilidade social dessas mulheres, como desemprego, baixo grau de escolaridade e histórico de abuso de drogas^33^. Entre as explicações correntes para a maior participação das mulheres cisgênero no tráfico de drogas, está a sua relação com parceiros íntimos ou familiares, estes já envolvidos com o tráfico^31^. Também há estudos que revelam uma escolha pessoal, em que a mulher teria a oportunidade de ganhar maior reconhecimento e *status* social em sua comunidade^34,35^.

Para mulheres trans e travestis, no entanto, há pouca investigação sobre o tema. O envolvimento das MTT com tráfico de drogas pode estar associado a maior prevalência de uso de drogas ilícitas por essa população^2,3^. Como o trabalho sexual não é considerado crime no país, a mera posse de drogas por MTT profissionais do sexo pode ser utilizada como motivo para a sua detenção. Ainda, o envolvimento com o tráfico pode ser devido a fatores como discriminação e preconceito, que culminam em falta de oportunidades de trabalho formal, podendo então o tráfico de drogas ser percebido por elas como uma potencial fonte de renda. Um estudo qualitativo conduzido com mulheres trans presas na Nicarágua e em El Salvador revelou que a falta de oportunidades de emprego formal foi o principal motivo reportado para que elas cometessem crimes^29^. Assim como nossos resultados, os motivos de prisão citados no estudo foram o tráfico de drogas, o roubo e a prostituição^29^.

O perfil de MTT com experiências de encarceramento evidencia a situação de maior vulnerabilidade social dessas mulheres trans e travestis. O estigma associado à transgeneridade^27^ pode ter implicações diretas nas condições de vida, como o baixo nível educacional, a difícil inserção no mercado de trabalho, a presença do trabalho sexual e falta de moradia, fazendo com que o encarceramento seja uma situação frequente. Apesar dos avanços legais no tratamento da população LGBTI+ privadas de liberdade, que confere às MTT os mesmos direitos reservados às mulheres cisgêneros, este artigo demonstra que o percentual de MTT com violação de direitos foi elevado, e ficam evidenciadas as dificuldades em se garantir de forma efetiva os direitos dessa população.

Diversas formas de violência, para além das ocorridas no ambiente carcerário, apresentaram altas prevalências na amostra estudada. Esses dados refletem a elevada incidência de violência baseada no gênero (VBG) enfrentada por MTT em diferentes contextos^36^. A VBG é um termo abrangente que engloba qualquer forma de violência física, sexual ou emocional perpetrada contra a vontade de uma pessoa e decorrente de desigualdades de poder fundamentadas em papéis de gênero^37^. Conforme destacado por Wirtz et al., a falha do Estado em reconhecer a identidade de pessoas trans e em protegê-las contra a discriminação contribui para a criação de ambientes propícios à ocorrência de violências, as quais, por vezes, são toleradas ou até mesmo normalizadas devido à ausência de sanções adequadas^36^.

O fato de o encarceramento de MTT ser em unidades femininas, masculinas ou em unidades específicas para a população LGBTI+, pode estar relacionado com as diversas situações de violência vivenciadas no cárcere. Apesar das robustas evidências de que a alocação de MTT em alas masculinas é prejudicial para a garantia do bem-estar físico e psicológico dessas detentas^4,6,7,9^, salientamos a necessidade de estudos que analisem a experiência de encarceramento de MTT com mulheres cisgênero ou em alas específicas para a população LGBTI+.

Para assegurar os direitos das mulheres trans e travestis privadas de liberdade no Brasil, torna-se imprescindível a implementação de iniciativas governamentais alinhadas com as diretrizes e normas preconizadas pela resolução do Conselho de Justiça. Ademais, são necessárias ações de políticas públicas destinadas a mitigar os contextos de vulnerabilidade que afetam essa população. Essas medidas incluem a implementação de políticas de cotas no ensino superior, a adoção de políticas afirmativas no mercado de trabalho para ampliar as oportunidades de emprego, a criação de políticas habitacionais e, por fim, o desenvolvimento de políticas que promovam uma maior equidade de gênero. Essas ações coletivas são cruciais para promover a inclusão, a proteção e a garantia dos direitos fundamentais dessa comunidade.

É importante ressaltar algumas limitações inerentes ao presente estudo. A principal delas está relacionada à dificuldade de estabelecer a relação temporal entre o encarceramento e outras situações vivenciadas pelas entrevistadas, como a situação sociodemográfica. Adicionalmente, dado que o foco principal da pesquisa não era o encarceramento, poucas questões sobre esse tema específico foram incluídas no questionário. A opção pela técnica de amostragem RDS, devido à natureza da população de difícil acesso, resulta em uma amostra por conveniência. Além disso, o estudo foi conduzido em cinco capitais brasileiras, o que não abrange todos os estados nem as MTT residentes em áreas rurais. Consequentemente, os resultados devem ser interpretados com cautela, não sendo passíveis de generalização para a totalidade da população de mulheres trans e travestis. Considerando a necessidade de compreensão mais abrangente, outros estudos investigando diversas experiências de MTT durante o encarceramento, especialmente em países latino-americanos, seriam valiosos para ampliar a compreensão desse panorama.
