# Supplementary material for: Erratum
Source: Rev Bras Epidemiol. 2024 Sep 30;27(Suppl 1):e240014.supl.1erratum. doi: 10.1590/1980-549720240014.supl.1erratum (PMC11494687; doi:10.1590/1980-549720240014.supl.1erratum)
Supplement: Related texts

# Correction: Errata

**DOI:** 10.1590/1980-549720240014.supl.1.2erratum

No artigo "**Perfil e experiências durante o encarceramento de mulheres trans e travestis (MTT) no Brasil: um estudo transversal**", DOI: https://doi.org/10.1590/1980-549720240014.supl.1.2, publicado no periódico Rev Bras Epidemiol. 2024; 27(Suppl 1): e240014.supl.1:

**Onde se lê:**

Maria Amélia de Sousa Mascena Vera

**Leia-se:**

Maria Amélia de Sousa Mascena Veras
